# A Probabilistic Model to Predict Clinical Phenotypic Traits from Genome Sequencing

**DOI:** 10.1371/journal.pcbi.1003825

**Published:** 2014-09-04

**Authors:** Yun-Ching Chen, Christopher Douville, Cheng Wang, Noushin Niknafs, Grace Yeo, Violeta Beleva-Guthrie, Hannah Carter, Peter D. Stenson, David N. Cooper, Biao Li, Sean Mooney, Rachel Karchin

**Affiliations:** 1 Department of Biomedical Engineering and Institute for Computational Medicine, The Johns Hopkins University, Baltimore, Maryland, United States of America; 2 Institute of Medical Genetics, School of Medicine, Cardiff University, Heath Park, Cardiff, United Kingdom; 3 Buck Institute for Research on Aging, Novato, California, United States of America; Accelrys, United States of America

## Abstract

Genetic screening is becoming possible on an unprecedented scale. However, its utility remains controversial. Although most variant genotypes cannot be easily interpreted, many individuals nevertheless attempt to interpret their genetic information. Initiatives such as the Personal Genome Project (PGP) and Illumina's Understand Your Genome are sequencing thousands of adults, collecting phenotypic information and developing computational pipelines to identify the most important variant genotypes harbored by each individual. These pipelines consider database and allele frequency annotations and bioinformatics classifications. We propose that the next step will be to integrate these different sources of information to estimate the probability that a given individual has specific phenotypes of clinical interest. To this end, we have designed a Bayesian probabilistic model to predict the probability of dichotomous phenotypes. When applied to a cohort from PGP, predictions of Gilbert syndrome, Graves' disease, non-Hodgkin lymphoma, and various blood groups were accurate, as individuals manifesting the phenotype in question exhibited the highest, or among the highest, predicted probabilities. Thirty-eight PGP phenotypes (26%) were predicted with area-under-the-ROC curve (AUC)>0.7, and 23 (15.8%) of these were statistically significant, based on permutation tests. Moreover, in a Critical Assessment of Genome Interpretation (CAGI) blinded prediction experiment, the models were used to match 77 PGP genomes to phenotypic profiles, generating the most accurate prediction of 16 submissions, according to an independent assessor. Although the models are currently insufficiently accurate for diagnostic utility, we expect their performance to improve with growth of publicly available genomics data and model refinement by domain experts.

## Author Summary

The Personal Genome Project (PGP) is an emerging community whose goal is to collect and publicly share genomes, environmental data, medical records, and clinical traits from tens of thousands of volunteers. This information may enable computer software to establish the relationships between patterns of alterations in human genomes and clinical phenotypic traits. We describe a novel, Bayesian mathematical model to predict such traits from genome sequence and population prevalence. The core of the model is a set of phenotypic penetrance estimates for aggregated genetic variants, which are learned without any information about particular individuals in a cohort of interest. We illustrate the model's utility in ranking individuals in the PGP cohort, according to their probability of having 146 phenotypes.

## Introduction

A central question in modern human genetics is how inter-individual variation impacts human phenotypes. Unprecedented technological advances will soon make whole genome DNA sequencing services available to a large number of people. However, interpreting the variant genotypes found in an individual's genome remains challenging, and is the focus of many academic, government, and commercial efforts. Here we address some limitations of state-of-the-art biomedical informatics tools to interpret genomic data, and we propose a Bayesian probabilistic model that begins to address these limitations.

An individual's whole genome sequence yields 3.2 million variant genotypes on average [1]. Genome interpretation requires reducing this very large number to a more tractable list. Current informatics tools prioritize variant genotypes, using database annotations, bioinformatics function prediction, and allele frequencies. For example, the PGP's GET-Evidence pipeline [1] prioritizes non-synonymous substitution variant calls over other alterations and ranks variant calls with a heuristic point system incorporating PolyPhen-2 classifications [2], and variant allele frequencies, variant and gene annotations in multiple public databases. The “Disease Risk of Volunteers Project” informatics pipeline identifies disease-causing mutations (DMs) in the Human Gene Mutation Database [3], eliminates any variants with minor allele frequency (MAF)>0.01, those predicted to be benign by two out of three bioinformatics classifiers, and those seen more than three times in their cohort. In both projects, short lists of putatively important risk variant genotypes identified by the pipelines are reviewed by researchers and shared with participants.

However, the purpose of personal genome interpretation is to understand how variant genotypes impact upon an individual's lifetime risk of specific diseases or traits. Annotating single variant genotypes is just the first step. Most human phenotypes result from a constellation of variant genotypes and non-genetic contributions. Here we shift the focus from interpretation of single variant genotypes to identifying genes and genotypes that impact the phenotype and estimating their penetrance. To our knowledge, the only previous comparable approach to this problem considered each variant genotype as an independent medical test with an associated likelihood ratio [4]. A “pre-test” probability of phenotype, based on age- and gender-based prevalence, was multiplied by a chain of likelihood ratios for each common variant, yielding a post-test probability of phenotype. In a pioneering study of the genome of a single individual, this method was used to predict the probability of 55 disease phenotypes [5]. The likelihood ratios were derived from extensive database annotations and 480 publications of cohort and case-control studies.

We present a formal Bayesian probabilistic model that for the first time integrates annotations of phenotype prevalence, both rare and common variant genotypes and disease-associated genes, and yields a single posterior probability for a phenotype of interest. We use self-reported phenotypes and medical information shared by participants in the PGP to quantitatively assess the performance of the model on a cohort of individuals. Notably, our models do not use information from the 130 members of the PGP cohort to fit or optimize parameters. However, eventually the availability of information from thousands of individuals could enable learning these parameters directly from individuals' genomes and reported phenotypes, enabling significantly better phenotype predictions.

## Results

**Figure 1:**
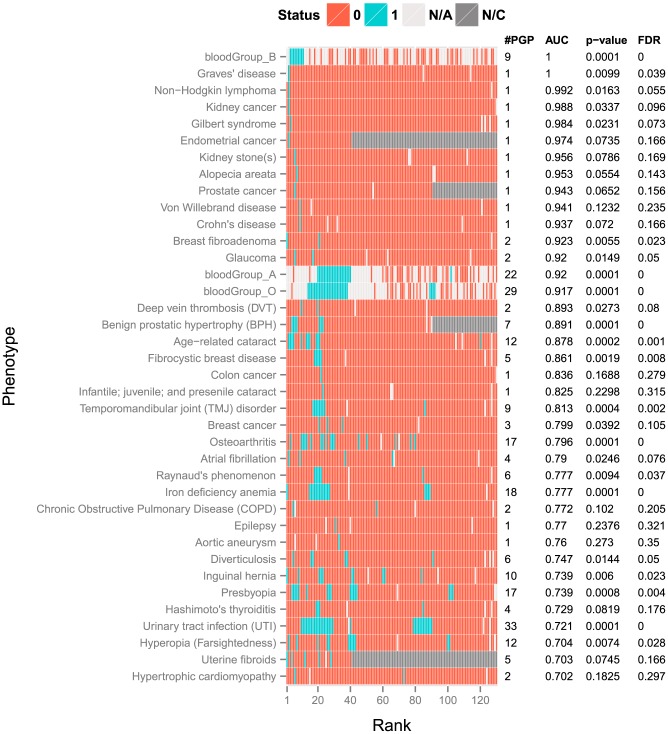
Prediction results of the model on 38 dichotomous phenotypes. Each row represents a clinical phenotype and consists of 130 cells, each of which represents a Personal Genome Project (PGP) participant. Cells in each row are ranked by the posterior probability that the participant has the phenotype. Cells are colored by true phenotypic status. Blue cells indicate that a participant has the phenotype, and red cells that a participant does not have the phenotype. If a cell is colored light grey, the true phenotypic status is unknown. If a cell is colored dark grey, the PGP participant is not considered in the evaluation because the phenotype is gender-specific. #PGP = number of participants in each row having the true phenotypic status. AUC = area under the receiver operating characteristic curve, a threshold-free metric of classifier performance. p-value and FDR = statistical significance of the AUC value, based on permutation testing.

For each phenotype, we used our model to compute the posterior probability of each individual having that phenotype (Text S1: Eq 3) and ranked the 130 PGP participants accordingly. The individual with the largest posterior probability was assigned Rank #1, the second largest Rank #2, and so forth. Then we assessed the ranking for each phenotype using area under the ROC curve (AUC) and computed the statistical significance of the AUC according to nominal p-value and false discovery rate (FDR) (Methods). Thirty-eight PGP phenotypes (26%) were predicted with area-under-the-ROC curve (AUC)>0.7, and 23 (15.8%) of these were statistically significant (p-value<0.05 and FDR<0.1) (Figure 1). Sixty-four phenotypes were predicted as random or worse (AUC≤0.5). Our statistics are based on the assumption that there is correlation structure among the phenotypes, which provides a small benefit to the number of significant predictions and expected FDR. If the phenotypes were truly uncorrelated, 21 predictions would be significant (p-value<0.05 and FDR<0.2) (Table S12).

**Figure 2:**
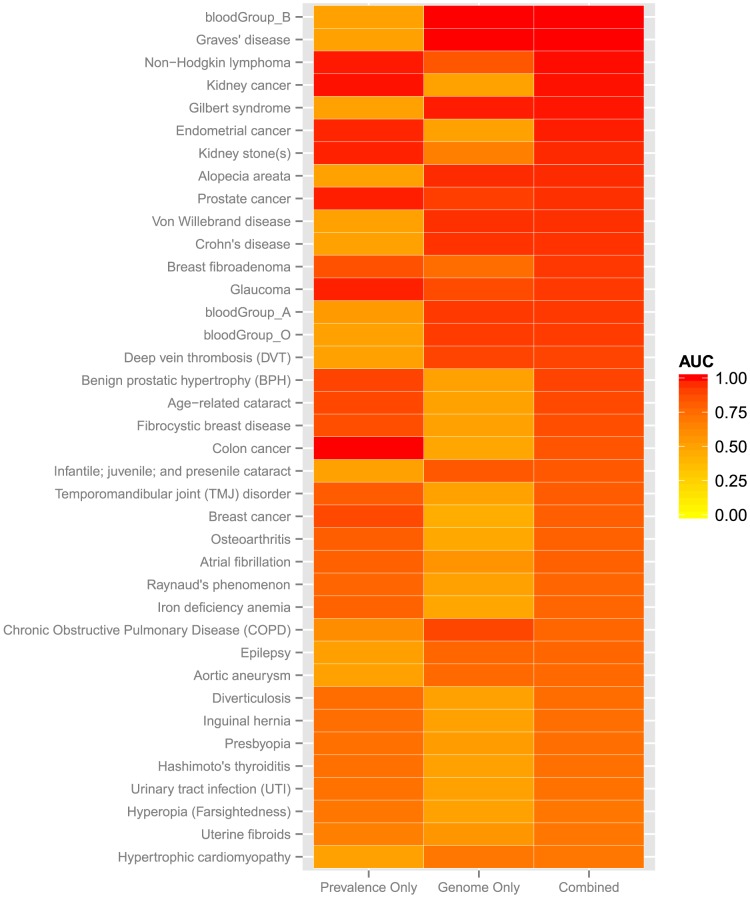
Contribution of population prevalence and genome sequence to prediction results in Fig. 1. Each row represents a phenotype and consists of three cells, representing (a) model predictions based only on phenotype-specific population prevalence (Prevalence Only), (b) model predictions based on genome sequence (with assumption that every phenotype and every individual has the same prevalence), and (c) model predictions that combine genome sequence and phenotype-specific population prevalence. Cells are colored by the area under the ROC curve (AUC) yielded by each model. Contributions vary among phenotypes due to differences in quality of available information with respect to prevalence and database annotations of variant genotypes.

The model incorporated both genome sequence and population phenotype prevalence, and we measured the contributions of each to prediction performance. First, AUC, p-values and FDR for the top predicted 38 phenotypes were computed using each individual's estimated phenotype prevalence instead of a posterior probability. Next, we repeated these computations using the genome sequences and assigning each phenotype the same baseline prevalence, set to be the average prevalence across all phenotypes. Comparison of genome-only, prevalence-only, and combined results showed that 14 phenotypes had higher genome-only than prevalence-only AUCs (Figure 2). Thus, these phenotypes likely have a strong genetic component, and at least some of the underlying genes and variant genotypes are represented in the annotation databases. Finally, we explored whether all categories of genomic annotations – GWAS hits, variant genotypes in disease-associated genes, and high-penetrance variant genotypes – were useful in predicting each phenotype, by calculating the prediction performance if only one of these had been used.

**Figure 3:**
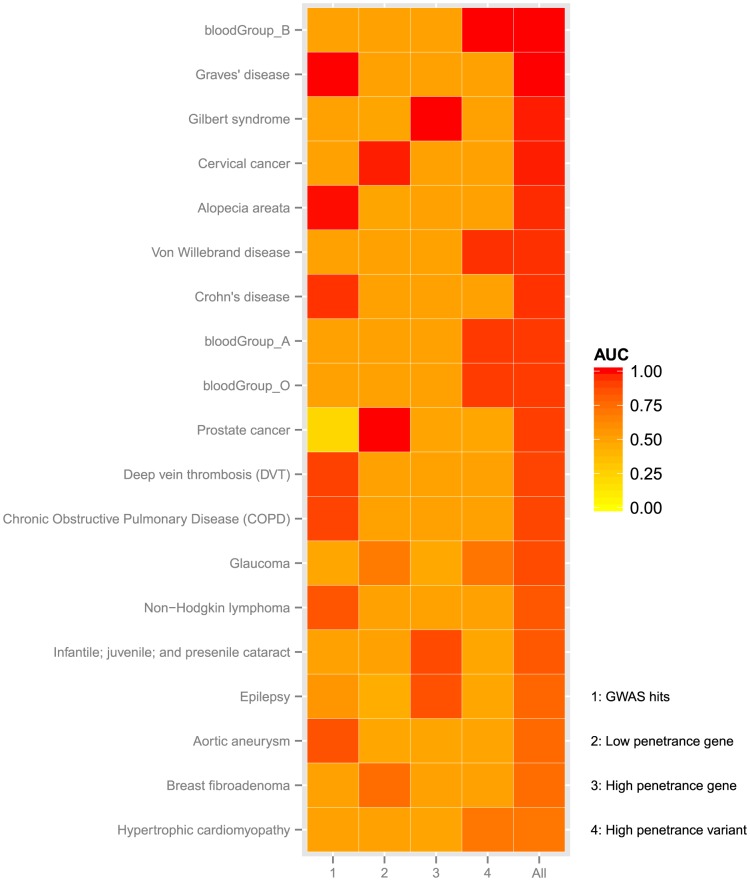
Contribution of GWAS hits, low penetrance genes, high penetrance genes, and high penetrance variants to prediction results. Each row represents a phenotype predicted with AUC>0.7 by genome sequence (Figure 2 (b)) and contains five cells. Cells are colored by the area under the ROC curve (AUC) yielded by a model that contains only 1:GWAS hits, 2:Low penetrance genes, 3:High penetrance genes, 4:High penetrance variants. The fifth cell shows AUC of the combination model used to assess results in this work that considers all of 1,2,3, and 4. The combination model generally yields the best performance: however, for most phenotypes, only one or two of 1, 2, 3 or 4 appears to contribute.

Six of the phenotypes were predicted best by GWAS hits – the autoimmune disorders Graves' disease, alopecia areata and Crohn's disease; the cardiovascular disorders, deep vein thrombosis and aortic aneurism; and chronic obstructive pulmonary disease (Figure 3). Only one PGP participant (PGP-48) had Graves' disease, and she was ranked second out of 130 (AUC = 1.0) (Figure 1). Her genome harbored numerous risk alleles at the sites of 16 GWAS hits (9 homozygous and 7 heterozygous risk alleles). One PGP participant (PGP-69) had alopecia areata (autoimmune-related hair loss), and he was ranked seventh out of 130 (AUC = 0.953). His genome harbored 7 GWAS hits (4 homozygous and 3 heterozygous risk alleles). A complete list of PGP participants with these six phenotypes and the underlying GWAS hits are in Tables S1, S2, S3, S4, S5, S6.

Three phenotypes were predicted best by non-synonymous coding variants in annotated disease genes and bioinformatics variant classifications – the common, hereditary liver disease Gilbert's syndrome, epilepsy and non-age-related cataracts (Figure 3). Only one of these predictions was statistically significant (Gilbert's syndrome, P = 0.023 and FDR = 0.073) (Figure 1). One PGP participant (PGP-125) reported having Gilbert's syndrome, and he was ranked third out of 130 (AUC = 0.984). He had a rare, heterozygous missense mutation P229L in the Gilbert's syndrome-associated gene *UGT1A1*. Of note, with only 130 samples, if only one PGP participant had a particular phenotype, statistical significance according to our permutation test required that the model allocate them rank 1–4 within the cohort.

Five phenotypes were predicted best by high penetrance variant genotypes – von Willebrand disease, hypertrophic cardiomyopathy, and three blood groups (Figure 3). Only the blood groups were statistically significant (Figure 1). The A, B and O blood groups were well represented in the 130 PGP participants, and known variant genotypes [6] ranked individuals with AUC = 0.92 for group A, AUC = 1 for group B, and AUC = 0.917 for group O. In addition, 27 phenotypes had combined results – genome sequence plus prevalence – better than or equal to prevalence-only AUC (Figure 2, Table S7).

With a few exceptions (blood groups and Gilbert's syndrome), all of our best-predicted phenotypes were complex and multi-genic. Common variants, likely involved in transcriptional regulation, and rare variants causing protein defects, both played important roles in these predictions. However, for each phenotype, the best predictions were generated by only a single category of annotations and were either GWAS hits, high penetrance variants, low penetrance genes containing rare variants, or high penetrance genes containing rare variants.

Of all the best-predicted phenotypes, only glaucoma benefited from more than one category of annotations – high penetrance variants and low penetrance genes. For this phenotype, the two PGP participants with glaucoma (PGP-15 and PGP-88) were ranked as 6 and 17 out of 130 (AUC = 0.92) (Figure 1). PGP-15 had a glaucoma-associated high-penetrance variant in the gene *WDR36* (A449T), and PGP-88 had a rare variant (N286T) in the glaucoma-associated gene *PCMTD1*.

In 2012–13, the Critical Assessment of Genome Interpretation (CAGI) blinded prediction experiment included a challenge based on prediction of PGP phenotypes. A total of 291 PGP participants provided phenotypic profiles, reporting their status with respect to 243 dichotomous clinical traits to the experiment organizers. We were one of several prediction teams, who were provided both genomic data for 77 PGP participants and 291 phenotypic profiles, of which 214 were decoys. The challenge was to identify the 77 PGP participants by matching their genomes and profiles. We used the posterior probabilities of our phenotypic models to provide a rank order matching of the PGP participants and their profiles. Briefly, for each participant, the phenotypic profiles were ranked from most probable to least probable for that individual. Prediction teams were evaluated by an independent assessor based on count of correct top-ranked profiles and also by mean rank of the correct profiles for all participants.

For 27 of the 77 PGP participants, genotypic data from 23andMe was also available to the prediction teams on the PGP website, and identification of these participants was considered to be trivial. Furthermore, the website contained the critical information that no blood or saliva samples had been collected for 108 of the profile decoys, thereby making it possible to exclude these profiles as potential matches. According to the independent assessor, after elimination of the 27 participants with genotypic data and the 108 profile decoys, our team correctly predicted the largest number (six) of top-ranked participants and had the lowest mean rank for correct profiles (25.4), of the 16 submissions to the challenge. Based on an empirical null distribution, our prediction had p-value<10^−4^ (Methods).

## Discussion

We introduce a Bayesian probabilistic model that allows individuals to estimate their risk of having a dichotomous phenotype. The models could be useful as an extension to existing pipelines for genome interpretation, such as those currently used by PGP (GET-Evidence) [1], DRV [7], UYG, and the Interpretome [8]. These pipelines rely on database annotations of variant genotypes and genes, allele frequencies and bioinformatics methods for variant function prediction. The PGP, DRV, and UYG pipelines yield lists of prioritized variant genotypes and associated evidence to support the hypothesis as to whether a single variant genotype is involved in a given disease/trait of interest. The Interpretome provides prioritized lists for rare variants and phenotype predictions based on common variants. Our Bayesian probabilistic model could use any of these prioritized lists and provide phenotype predictions, which consider the contributions of both rare and common variants.

### Strengths of our model

The model presented here could be used in the setting of an adult volunteer cohort. Within this setting, it provides interpretable results to help individuals understand their risk of a phenotype of interest. To our knowledge, it is the first such model to use population-level prevalence as a prior, integrate the contribution of rare and common variant genotypes harbored by an individual, and consider the modulating effects of incomplete penetrance, environmental, and unknown factors. In addition to a final posterior estimate of an individual's phenotypic risk, the model provides information about the separate contributions of population-level prevalence and personal genome sequence. Each individual can also learn their rank probability within the cohort, a number that may be easier to understand than a raw posterior probability. We can further inform individuals as to how much each prediction can be trusted, based on the model's previous performance.

The model is flexible, and it can be reasonably applied to predict any individual's probability of having any dichotomous phenotype with a genetic component. The key elements are: estimating prior probabilities that the individual has the phenotype, ideally considering age, gender and ancestry; identifying annotated genes and variant genotypes associated with the phenotype; finding the subset of those present in the individual's genome sequence and estimating their aggregate penetrance; and finally computing the posterior probability that the individual has the phenotype. Genes and variant genotypes are sorted into four categories: low penetrance variants, low penetrance genes, high penetrance variants, and high penetrance genes. The aggregate penetrance of each category is estimated with a mathematical model (Text S1: Eqs. 10–14). Bioinformatics variant function predictions are also incorporated. Variant genotypes in all phenotype-associated genes are scored with VEST [9], a bioinformatics classifier that estimates a significance level (p-value) for each variant score. The p-values are aggregated into a gene-level score using Fisher's method, then used to estimate the posterior probability that the gene was affected, with empirical data (Methods). Any variant function prediction method that yields p-values and/or any of a number of gene-level variant aggregation methods can be used.

The advantage of integrating the impact of both rare and common variants can be quantified by comparing our model with a model based only on the burden of putatively damaging alleles (MAF<0.01) in our sets of phenotype-associated genes. When applied to the same PGP cohort, this simple burden model yielded only one predicted phenotype that was statistically significant after multiple testing correction (in contrast to our model's 23 statistically significant predicted phenotypes) (Figure S1).

### Limitations of the current model

Incomplete and inaccurate information about genes, variant genotypes, and phenotypes in current databases limit the model's utility. As an example, for 42 PGP phenotypes, we were unable to find any associated genes or variants. Furthermore, the association of a particular gene or variant to a phenotype may not be quantitative, with respect to effect size. Thus, we make simplifying quantitative assumptions about their aggregate penetrance, as follows: 1) GWAS hits and any disease-gene associations lacking careful curation are assigned low penetrance; 2) curated (DM) disease variants and genes in HGMD and OMIM are assigned high penetrance; 3) presence of a highly penetrant variant dominates the posterior (Text S1: Eq 4); 4) penetrance of a GWAS hit is estimated by its reported odds ratio, allele frequency and phenotype prevalence (Text S1: Eqs. 21–25); 5) the effect sizes of rare non-silent variants are assigned to be greater than the effect sizes of GWAS hits associated with the same phenotype [10]; 6) changes in gene product function are computed using only rare (MAF<0.01) non-silent variants; 7) interactions among genes and variant genotypes are not considered; 8) low prevalence is assigned to variants and genes with high penetrance; 9) Only small-scale, non-silent variants are considered, although some phenotypes may be better predicted by other genetic or epigenetic alterations.

**Figure 4:**
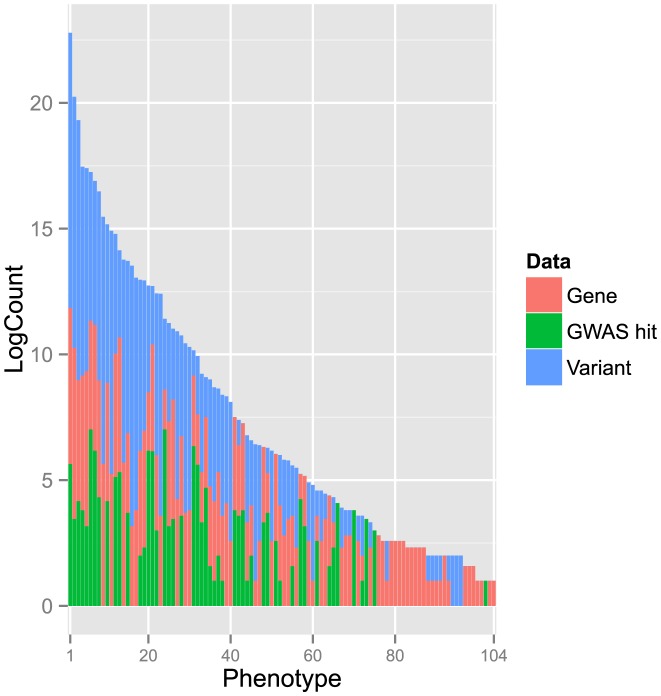
Distribution of annotated genes, GWAS hits and high penetrance variants for phenotypes analyzed in this study. Phenotypes are ordered by total counts of annotated genes and variants that we found. Counts are shown on a log scale for easier visualization. For each phenotype the (log) count of annotated genes is colored red, GWAS hits green, and high penetrance variants blue. Some phenotypes have a very large number of annotations and others have very few. For 42 phenotypes, we did not find any annotated genes or variants.

The phenotypes predicted in our study include those known to have strong genetic components, such as Gilbert's syndrome, von Willebrand disease and epilepsy [11]–[13] and others lacking evidence of strong genetic contribution, such as hiatal hernia and dental cavities [14], [15]. Of 146 phenotypes, we identified associated genes or variant genotypes for 104. If we consider the raw count of annotated genes, GWAS hits, and high penetrance variants per phenotype, the range is large, with some phenotypes having thousands and others fewer than ten annotations (Figure 4). These differences affected our ability to predict phenotypes. For example, Gilbert's syndrome and von Willebrand disease were among our best-predicted phenotypes. Both have been studied for many years and causal genes (*UGT1A1* for Gilbert's syndrome and *VWF* for von Willebrand disease) are known [11], [12]. By contrast, ulcerative colitis is believed to have a genetic component but the causal genes are still largely unknown [16], and no genetic components for dandruff have been identified [17].

Out of seven cancer phenotypes, only kidney cancer and non-Hodgkins lymphoma were predicted with AUC>0.7 and high statistical significance (P-value<0.05, FDR<0.1), and these predictions were driven by population-based priors (Figure 2). Because most cancers have strong environmental contributions, improved predictions would require more information about carcinogen exposures and resulting patterns of somatic mutations. Our current models rely on germline variants, which may be useful for predicting familial cancers, but are less relevant for the more common sporadic cancers.

Our model predictions for 64 PGP phenotypes were no better than random (Table S8). For 38 phenotypes, either we were unable to find evidence of phenotypic association with genes or variant genotypes, or we found such evidence but were unable to match it with variants in any PGP genomes. For the remaining 26 phenotypes, we suspect that errors in annotations and in our model assumptions about penetrance are responsible. For example, our predictions of hereditary neuropathies in PGP (including Charcot-Marie-Tooth disease) yielded an AUC of 0.405. We identified 813 mutations in 37 genes associated with this phenotype in HGMD's high confidence of disease association (DM) class. Although six of these were found in the genomes of nine PGP participants, none of them reported this clinical phenotype. It appears that in assuming that HGMD DM mutations had high penetrance, we overestimated the probability that these nine individuals had a hereditary neuropathy. In addition, one PGP participant reported the phenotype but did not have any of the mutations, which could be due to our omission of the most common causes of hereditary neuropathy – duplications or deletions of the *PMP22* (peripheral myelin protein) gene [18].

### Future work

Improvements in the infrastructure of disease gene annotations and the growing communities of adult volunteers, such as the PGP, have the potential to significantly improve the utility of the model proposed here. We have discussed the many simplifying assumptions about penetrance parameters that were used in the current work. However, if a resource that provided the genomes and phenotypic profiles of a large number of people were available, we could use it for maximum likelihood estimation of the penetrance parameters in our model. Such a resource would also allow us to generate reference panels for adult genetic testing. We could use our model to compute the posterior probability of each sequenced individual for each phenotype of interest and generate ranked lists consisting of thousands of individuals. As the lists grow larger, they would also grow in utility for individuals who learn their ranking within the lists. The model could also be extended to include genomic copy number variations and even data from microbiomes.

We expect that as a larger number of individuals become interested in personal genomics, members of communities such as the PGP will have access to family pedigree information and/or genotype or sequencing data from family members. The availability of pedigree information would allow us to estimate a personalized phenotype prior for each individual, rather than estimating these priors only by population prevalence. Numerous methods have been developed for this purpose [19]–[21]. Genotype or sequencing data from family members could be used to improve both imputation of missing genotypes and phasing [22], [23]. While phasing is not currently considered in our models, knowledge of whether multiple variants are in the same haplotype or simply on the same chromosome could be informative with respect to their phenotypic impact [24]–[26].

We are optimistic that integrated models such as the one presented here will contribute to increasingly accurate and interpretable predictions of clinical phenotype from genome sequence in the near future.

## Methods

### PGP genomes

We downloaded variant genotypes from 174 genomes sequenced by Complete Genomics with the 2.0 Standard pipeline, from the PGP website (http://my.pgp-hms.org) (as of 02/10/2014). The PGP genomic data were obtained by sequencing DNA purified from lymphocyte cell lines [1]. Variant genotypes were obtained from the GFF format file produced by PGP's Genome-Environment-Trait-Evidence (GET-Evidence) pipeline [1]. Only variant position, reference and alternative allele calls from the GFF file were employed. 44 genomes were excluded from consideration due to missing either a trait survey, associated age, gender or ancestry, or did not have GET-Evidence GFF files, yielding 130 genomes to be analyzed.

### PGP-phenotypes

PGP participants have the option of filling out a “traits questionnaire”, consisting of 239 dichotomous phenotypes. Blood groups were also provided in “Personal Health Records” of the participants, yielding a total of 243 phenotypes. Results of the questionnaire and blood groups were downloaded from the PGP website and considered to be accurate. Of the 243 phenotypes, only 153 were reported by at least one PGP participant, and 146 also had available prevalence information.

### Phenotype prevalence and heritability

Internet searches for information about the prevalence and heritability of each trait were performed manually. Wherever possible, we found the most relevant prevalence for an individual, considering her/his age, gender, and self-reported ancestry. Data sources included SEER (NCI), websites for CDC (http://www.cdc.gov) and HHS (http://www.hhs.gov/), and the published literature. A complete list of sources and prevalence estimates is to be found in Table S9. Scripts are available on request from the authors.

### Gene and variant annotations

Variant annotations were collected from NHGRI-GWAS (https://www.genome.gov/26525384) (downloaded 09/11/2013), HGMD Professional (HGMD Pro) v.2013,2 [3] (downloaded 06/26/2013), and SNPedia [27]. Gene annotations were collected from OMIM [28] (downloaded 09/09/2013), disease-gene associations were mined from the literature (http://diseases.jensenlab.org downloaded 07/25/2013), and HGMD Pro v.2013.2 [3]. NHGRI GWAS variants were included if they had an odds-ratio (OR) or beta regression coefficient >1 and < = 20. HGMD Pro variants were included if and only if they were in the most confident disease mutation class (DM). SNPedia was used as to identify SNPs associated with blood groups, known to be high penetrance (http://snpedia.com/index.php?title=ABO_blood_group&oldid=560223) [27]. Disease-gene associations mined from literature were included only if they were rated as high confidence by the mining algorithm. For associations from Jensen's database [29], which computes a Z-score for each disease-gene association, we required Z-score >4.0 or ranking in the top-5 associated genes for the disease, according to Z-score. HGMD Pro genes were considered to be in the DM class if they contained at least one mutation in the DM class. All HGMD Pro annotations used in the models are included in Table S10. All GWAS annotations and their allele frequencies are in Table S11.

### Probabilistic model

**Figure 5:**
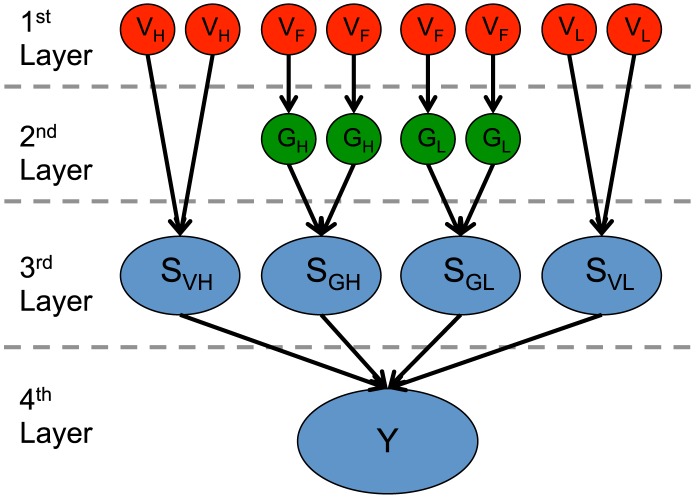
Topology of the model to predict phenotype from an individual's genome sequence. Red nodes in the first layer of the model represent the individual's genotype calls at genomic positions associated with the phenotype of interest. They are sorted into three categories: *V_H_* (HGMD DM variants), *V_L_* (NHGRI GWAS hits), and *V_F_* (<0.01 MAF in any population reported in ESP6500 (http://evs.gs.washington.edu/EVS/) or 1000 Genomes [30]), found in genes annotated as associated with the phenotype. Green nodes in the second layer represent genes split into high penetrance G_H_ or low penetrance G_L_ based on database annotations. Blue nodes in the third layer are Bernoulli random variables, abstractly representing mechanisms that explain the phenotype, sorted into those altered by high penetrance variants S_VH_, low penetrance variants S_VL_, high penetrance genes S_GH_, or low penetrance genes S_GL_. The blue node *Y* is a Bernoulli random variable representing individual phenotypic status. Directed edges show the dependencies between nodes. A set of model parameters is estimated for each phenotype and each individual.

We designed a Bayesian model to predict whether an individual had a phenotype of interest, based on genome sequence and estimated prevalence (Figure 5). Three categories of variable are included in the model. Categorical variables (0, 1, or 2) in the first layer represent observed genotypes, limited to those with phenotype-associated variants and predicted functional variants. Real-valued variables [0,1] in the second layer represent the probability that phenotype-associated genes are functionally altered. To estimate the aggregated penetrance of the genotypes, functional alterations are grouped into four abstract categories in the third layer. The probability that each of these categories is altered depends either on high penetrance variants (Bernoulli variable S_VH_); low penetrance variants (Bernoulli variable S_VL_); high penetrance genes (Bernoulli variable S_GH_); or low penetrance genes (Bernoulli variable S_GL_). The joint distribution of S_VH_, S_VL_, S_GH_, S_GL_ is used to infer the state of Bernoulli variable *Y*, which represents phenotype status. (All equations and derivations are in Text S1).

### Model assessment

Each phenotypic model was assessed by its ability to correctly rank individuals in the PGP cohort, as area under the ROC curve (AUC). No cross-validation was performed because neither model topology nor parameters were estimated or optimized with information from the PGP cohort. P-values and FDR were estimated with permutation. We applied two permutation tests. In the first test, the identities of PGP participants were shuffled, and in the second test, phenotype labels were shuffled. The first test preserves correlation structure among phenotypes within each participant. The second test assumes that phenotypes are independent and exchangeable. (Mathematical details are in Text S1).

### Predicted functional impact on gene products

We used the Variant Effect Scoring Tool (VEST) [9] to score the functional impact of variants and combined the VEST p-values, using Fisher's method, yielding a gene-level VEST statistic, denoted as *T_GENE_* (Text S1: Eq. 2). We derived the probability that the gene was functionally altered by all rare variants observed in the individual, denoted as *P*(*G* = 1|*T_GENE_*) using Bayes Rule (Text S1: Eq. 24). All VEST scores and p-values are included in Table S13.

### Rank order matching of PGP participants and phenotypic profiles in CAGI 2012–13

For each of the 77 PGP participants in the CAGI challenge, we used their genome sequence as input to models for each of the 243 phenotypes included in the challenge, and the posterior probability of each phenotype was computed. The match between each PGP genome and phenotypic profile was modeled with a Bernoulli likelihood, and the probability of each matched pair was calculated. Profiles were ranked from most to least probable. (Text S1)

### Assessment of phenotype-genotype matching algorithms in CAGI 2012–13

Prediction accuracy was measured by an independent assessor with the following criteria. First, the number of correctly top-ranked phenotypic profiles was computed. To assess the significance of that finding, benchmark or null prediction used uniformly random matches between phenotypic profiles and genomes, i.e., for a given genome, each phenotypic profile being equally possible. The simulation was repeated 10^4^ times and the number of correctly top-ranked profiles was recorded each time. In this setting, none of the simulations yielded five or more correctly top-ranked phenotypic profiles to the corresponding genomes, and hence the significance level for observing five or more correct matches is <10^−4^.

Software to implement all methods is available from authors on request.

## Supporting Information

Figure S1 **Prediction results of simple mutation burden model.** Six phenotypes predicted with AUC>0.7 are shown. Each row represents a clinical phenotype and consists of 130 cells, each of which represents a Personal Genome Project (PGP) participant. Cells in each row are ranked by the burden of putatively damaging alleles (MAF<0.01) in the same sets of phenotype-associated genes used in Figure 1. Cell coloring has the same meaning as in Figure 1. #PGP = number of participants in each row having the true phenotypic status. AUC = area under the receiver operating characteristic curve, a threshold-free metric of classifier performance. p-value and FDR = statistical significance of the AUC value, based on permutation testing.
(PDF)

Table S1 **GWAS hits correctly identified that PGP-48 has Graves' disease.** A single PGP participant had Graves' disease and she was ranked second out of 130, according to the posterior probability of having this phenotype (AUC = 1.0, P-value = 0.01, FDR = 0.039). Listed are the rsIDs of 16 GWAS hits, the risk alleles harbored by PGP-48, and the zygosity of each GWAS hit.
(XLSX)

Table S2 **GWAS hits correctly identified that PGP-69 has alopecia areata.** A single PGP participant had alopecia areata, and he was ranked seventh out of 130, according to posterior probability (AUC = 0.953, P-value = 0.055, FDR = 0.143). Listed are the rsIDs of 7 GWAS hits, the risk alleles harbored by PGP-69, and the zygosity of each GWAS hit.
(XLSX)

Table S3 **GWAS hits correctly identified that PGP-39 has Crohn's disease.** A single PGP participant had Crohn's disease, and she was ranked ninth out of 130, according to posterior probability (AUC = 0.937, P-value = 0.072, FDR = 0.166). Listed are the rsIDs of 97 GWAS hits, the risk alleles harbored by PGP-39, and the zygosity of each GWAS hit.
(XLSX)

Table S4 **GWAS hits correctly identified that PGP-142 and PGP-72 have deep vein thrombosis.** Two PGP participants had deep vein thrombosis, and they were ranked tenth and twentieth out of 130, according to posterior probability (AUC = 0.893, P-value = 0.027, FDR = 0.08). Listed are the rsIDs of 9 GWAS hits, the risk alleles harbored by PGP-142 and PGP-72, and the zygosity of each GWAS hit.
(XLSX)

Table S5 **GWAS hits predicted that PGP-158 has aortic aneurism.** One PGP participant had aortic aneurism, and she was ranked 34 out of 130, according to posterior probability (AUC = 0.76, P-value = 0.273, FDR = 0.35). Listed are the rsIDs of 2 GWAS hits, the risk alleles harbored by PGP-158, and the zygosity of each GWAS hit.
(XLSX)

Table S6 **GWAS hits correctly identified that PGP-39 and PGP-38 have chronic obstructive pulmonary disease (COPD).** Two PGP participants had COPD, and they were ranked fifth and 56th out of 130, according to posterior probability (AUC = 0.772, P-value = 0.102, FDR = 0.205). Listed are the rsIDs of 6 GWAS hits, the risk alleles harbored by PGP-39 and PGP-38, and the zygosity of each GWAS hit.
(XLSX)

Table S7 **Phenotype model prediction performance (AUC) for 130 PGP participants, using genome sequence only, prevalence only, and both.** Phenotypes are sorted by the difference between AUC of the prevalence only model and the AUC of the model that uses both genome sequence and prevalence. Only phenotypes reported in at least one PGP are listed.
(XLSX)

Table S8 **Phenotypes that were predicted no better than random by our models. **Phenotype: the poorly predicted clinical phenotypes. #PGP: the number of PGP participants who reported having the phenotype. AUC: the area under the receiver operating characteristic curve of our model predictions. p-value: the statistical significance of each AUC. FDR: the false discovery rate (FDR). None of the predictions are significant.
(XLSX)

Table S9 **Prevalences for each phenotype used in this work, based on age, gender, and ancestry when available.** Phenotypes are listed in column A. Columns B–H list seven ancestral populations for which we were able to establish phenotype population prevalence. For each ancestral population, phenotype prevalence is listed for each combination of gender and age range. Column I shows the source(s) of the data for the phenotype.
(XLSX)

Table S10 **Annotations of HGMD variants and genes.** Phenotypes are listed in column A. For variants, columns B–H list genomic location, reference and risk alleles, PubMed identifiers, and mutation consequence type. For genes, column B lists HUGO identifier.
(XLSX)

Table S11 **Annotations of GWAS hits.** Phenotypes are listed in column A. Columns B–H list genomic location, reference and risk alleles, allele frequency, and reported effect size (odds ratio).
(XLSX)

Table S12 **Phenotypes predicted with significant AUC using a null model that assumes phenotypes are independent and exchangeable.** If no correlation structure existed among the phenotypes, statistical significance could be assessed by shuffling phenotype labels with respect to their predicted probabilities. In this case, the model's performance declines slightly, yielding 21 phenotypes with P-value<0.05 and FDR<0.2. #PGP = number of participants in each row having the true phenotypic status. AUC = area under the receiver operating characteristic curve, a threshold-free metric of classifier performance. P-value and FDR = statistical significance of the AUC value, based on permutation test shuffling the phenotype labels.
(XLSX)

Table S13 **Variant Effect Scoring Tool (VEST) scores.** All variants scored by VEST are shown. Columns A–L list genomic location, strand, reference and alternative alleles, HUGO identifiers, amino acid substitutions, VEST score, VEST p-value, and MAF in European-American (EA), African-American (AA) populations (from Exome Variant Server), and 1000 genomes database where available.
(XLSX)

Text S1 **Supplementary material.**
(DOCX)
