# Supplementary figures and images for: A Probabilistic Model to Predict Clinical Phenotypic Traits from Genome Sequencing
Source: PLoS Comput Biol. 2014 Sep 4;10(9):e1003825. doi: 10.1371/journal.pcbi.1003825 (PMC4154636; doi:10.1371/journal.pcbi.1003825)

Phenotype

Status 0 1 N/A N/C

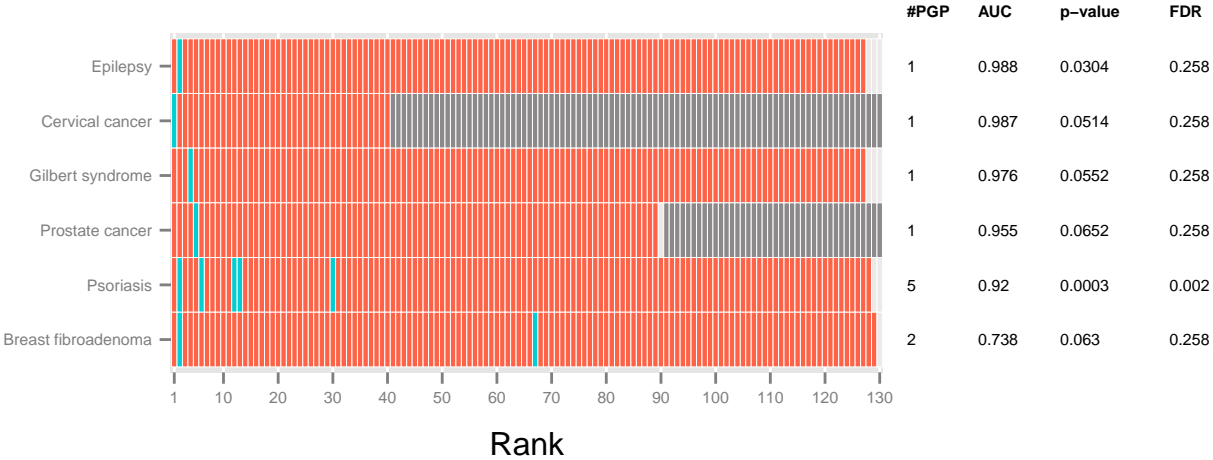

Supplement: Figure S1 — Prediction results of simple mutation burden model. Six phenotypes predicted with AUC>0.7 are shown. Each row represents a clinical phenotype and consists of 130 cells, each of which represents a Personal Genome Project (PGP) participant. Cells in each row are ranked by the burden of putatively damaging alleles (MAF<0.01) in the same sets of phenotype-associated genes used in Figure 1. Cell coloring has the same meaning as in Figure 1. #PGP = number of participants in each row having the true phenotypic status. AUC = area under the receiver operating characteristic curve, a threshold-free metric of classifier performance. p-value and FDR = statistical significance of the AUC value, based on permutation testing. (PDF) [file pcbi.1003825.s001.pdf]
