# Supplementary material for: A Probabilistic Model to Predict Clinical Phenotypic Traits from Genome Sequencing
Source: PLoS Comput Biol. 2014 Sep 4;10(9):e1003825. doi: 10.1371/journal.pcbi.1003825 (PMC4154636; doi:10.1371/journal.pcbi.1003825)
Supplement: Text S1 — Supplementary material. (DOCX) [file pcbi.1003825.s015.docx]

**Supplementary Material**

**Mathematical Details**

**Predicted functional impact of variants on gene products**.

We predicted the impact of variants on the protein product of a gene, in a particular individual, for all genes annotated as associated with the phenotype. Only rare variants were considered (MAF < 1% in ESP6500 and 1000 Genomes). Then, each rare variant that caused an amino acid substitution was scored with the Variant Effect Scoring Tool (VEST) [[1](#_ENREF_1)], yielding a score *m_i_*. Rare truncating (nonsense, nonstop, frameshift) and splice site variants *d_j_* were assumed to have on average a larger impact than rare missense variants. These events were given a score proportional to the highest scoring amino acid substitution variant in the gene and their allele frequency *AF_dj_* in the 130 PGP genomes as

$$d_{j}=\max_{i}\{m_{i}\}\times(1-AF_{d_{j}})$$

(Eq. 1)

We made the simplifying assumption that rare variants in a gene were not in linkage disequilibrium and were therefore independent. We used Fisher’s method to combine their VEST p-values, yielding a gene-level VEST statistic

$$T_{GENE}=-2\times\sum_{i=1}^{N} \ln(p_{i})$$

(Eq. 2)

We derived the probability $P(G=1|T_{GENE})$ that the gene was functionally altered by all rare variants observed in the individual, using Bayes Rule (Eq. 24).

**Topology of the probabilistic model.**

The model has the same overall topology, irrespective of the phenotype predicted and the individual being assessed (Figure 5).

*First layer*. The nodes in the first layer represent observed genotypes (0, 1 or 2) from an individual’s genome (homozygous reference allele, heterozygous allele, or alternate homozygous allele). Only genotypes annotated as directly associated with the phenotype are included. Genotypes are sorted into the following categories: high penetrance *V_H_* (HGMD DM variants); low penetrance *V_L_* (NHGRI GWAS hits); and rare (putatively functional) genotypes *V_F_* (<0.01 MAF in any population reported in ESP6500 [[2](#_ENREF_2)] or the 1000 Genomes Project [[3](#_ENREF_3)]). Putatively functional genotypes are only counted if they occur in genes annotated as being associated with the phenotype.

*Second layer.* These nodes represent genes, split into those annotated as high penetrance G_H_ or low penetrance G_L_. Their values depend on links to nodes in the first layer. Only genes whose translated products were bioinformatically predicted to be functionally altered by V_F_ genotypes are included (Materials and Methods: Predicted functional impact on gene products and Eq. 24).

*Third layer*. These nodes are Bernoulli random variables, which represent sets of hidden mechanisms that account for the clinical phenotype. Conditional independence given an individual's genomic data is assumed. The probability that each of the nodes is set to 1 depends on the high penetrance variants (Bernoulli variable S_VH_); the low penetrance variants (Bernoulli variable S_VL_); the high penetrance genes (Bernoulli variable S_GH_); and the low penetrance genes (Bernoulli variable S_GL_), respectively. The joint distribution of S_VH_, S_VL_, S_GH_, S_GL_ is used to infer the state of Bernoulli variable *Y.*

*Fourth layer.*  The Bernoulli variable *Y* represents the phenotypic status of the individual, and the posterior probablity of *Y* is the final output of the model.

**Inference of phenotype status.**

The topology of the model yields the following equation for the posterior probability of an individual's phenotypic status, given genome sequence data.

(Eq 3)

where the summation is over all possible configurations of *S_VH_, S_GH_, S_GL_* and *S_VL_.*

We reduce the number of penetrance parameters by assuming that we can disregard lower penetrance genotypes if higher penetrance genotypes are present, as follows:

(Eq 4)

Using (Eq 4), (Eq 3) can be rewritten so that the joint distribution of S_VH_, S_VL_, S_GH_, S_GL_ depends only on nine parameters.

$$\begin{matrix} P(Y=1|Data)= \\ P(Y=1|S_{VH}=1)P(S_{VH}=1|Data) \\ +P(Y=1|S_{VH}=0,S_{GH}=1)P(S_{VH}=0,S_{GH}=1|Data) \\ +P(Y=1|S_{VH}=S_{GH}=0,S_{GL}=1)P(S_{VH}=S_{GH}=0,S_{GL}=1|Data) \\ +P(Y=1|S_{VH}=S_{GH}=S_{GL}=0,S_{VL}=1)P(S_{VH}=S_{GH}=S_{GL}=0,S_{VL}=1|Data) \\ +P(Y=1|S_{VH}=S_{GH}=S_{GL}=S_{VL}=0)P(S_{VH}=S_{GH}=S_{GL}=S_{VL}=0|Data) \end{matrix}$$

(Eq 5)

The posterior probabilities of S_VH_, S_VL_, S_GH_, S_GL_ are computed as:

$$P(S_{VH}=1|Data)=\left\{\begin{matrix} 1,\text{ annotated variant genotype match} \\ 0,\text{ otherwise} \end{matrix}\right.$$

(Eq. 6)

$$P(S_{GH}=1|Data)=\max\left\{\begin{matrix} \max_{i}\{P(G_{H_{i}}=1|Data)\}-\max_{i}\{E[P(G_{H_{i}}=1)]\} \\ 0 \end{matrix}\right.$$

(Eq. 7)

where $P(G_{H_{i}}=1|Data)$ is calculated as in (Eq. 24) and *Data* is the VEST gene level statistic (Eq. 2). We make the simplifying assumption that if there are multiple high penetrance genes, the gene with maximum $P(G_{H_{i}}=1|Data)$ dominates, if it exceeds a baseline.

$$P(S_{GL}=1|Data)=1-\left[\prod_{i} (1-P(G_{L_{i}}=1|Data))\right]^{\alpha_{S_{GL}}}$$

(Eq. 8)

where $P(G_{L_{i}}=1|Data)$ is calculated as in (Eq. 24) and *Data* is the VEST gene level statistic (Eq. 2). If there are multiple low penetrance genes, the combined impact of $P(G_{L_{i}}=1|Data)$ is estimated with a noisy-or model [[4](#_ENREF_4)], exponentiated by a phenotype-specific weight ($\alpha_{S_{GL}}$), which controls for ascertainment bias (some phenotypes have hundreds of annotated low-penetrance genes while others have very few annotated low-penetrance genes) (Eq. 15).

$$P(S_{VL}=1|Data)=1-\left[\prod_{i} OR_{i}^{V_{L_{i}}}\right]^{-\alpha_{S_{VL}}}$$

(Eq. 9)

where OR_i_ is the odds ratio of genotype $V_{L_{i}}\in\{0,1,2\}$. Ascertainment bias is controlled with a phenotype-specific weight ($\alpha_{S_{VL}}$) (Eq. 17).

The penetrances of S_VH_, S_VL_, S_GH_, S_GL_ are computed as:

$$P(Y=1|S_{VH}=1)=\left\{\begin{matrix} 0.90\text{, Homozygous variant genotype or dominant heterozygous genotype} \\ 0.45\text{, Heterozygous genotype with unknown genetic model} \end{matrix}\right.$$

(Eq. 10)

In the absence of quantitative annotations (effect size) we estimate that a homozygous variant genotype or heterozygous variant genotype (if the genetic model is dominant) has penetrance of 0.9 and a heterozygous variant genotype has penetrance of 0.45, when the genetic model is unknown to us. For one “high penetrance variant” phenotype -- blood type -- we used information from SNPedia [[5](#_ENREF_5)] to estimate penetrance based on genotype of SNPs rs8176719, rs8176746, and rs8176747.

$$P(Y=1|S_{VH}=S_{GH}=S_{GL}=0,S_{VL}=1)=\frac{1}{n}\sum_{i=1}^{n} P(Y=1|V_{i}=1)$$

(Eq. 11)

where $P(Y=1|V_{i}=1)$ is computed by (Eq. 23) and *n* is the total number of low penetrance variants associated with the phenotype.

$$P(Y=1|S_{VH}=0,S_{GH}=1)=\frac{q\times P(Y=1)}{P(V=1)}$$

(Eq. 12)

where *q* is a variable related to $k_{1}\times\frac{1}{n}\sum_{i=1}^{n} OR_{i}$ (Eqs. 20-23), P(Y=1) is the prevalence of the phenotype for the individual, and P(V=1) is the frequency of a rare variant, estimated as 0.01. $k_{1}=5$ based on estimates by [[6](#_ENREF_6)] about the higher penetrance of rare vs. common variants.

$$P(Y=1|S_{VH}=S_{GH}=0,S_{GL}=1)=\frac{q\times P(Y=1)}{P(V=1)}$$

(Eq. 13)

where *q* is a variable related to $k_{2}\times\frac{1}{n}\sum_{i=1}^{n} OR_{i}$ (Eqs. 20-23), P(Y=1) is the prevalence of the phenotype for the individual, and P(V=1) is the frequency of a rare variant, estimated as 0.01. $k_{2}=2$ based on estimates by [[6](#_ENREF_6)] about the higher penetrance of rare vs. common variants.

$$P(Y=1|S_{VH}=S_{GH}=S_{GL}=S_{VL}=0)=\frac{[5]}{E[P(S_{VH}=S_{GH}=S_{GL}=S_{VL}=0)]}$$

(Eq. 14)

Derivation of Eq. 14

$$\begin{matrix} & \text{Prevalence} & =E[P(Y=1)]=\sum P(Y=1|Data)P(Data) \\ & & =\sum P(Y=1|S_{VH},S_{GH},S_{GL},S_{VL})\times E[P(S_{VH},S_{GH},S_{GL},S_{VL})] \\ \text{[1]} & & =P(Y=1|S_{VH}=1)\times E[P(S_{VH}=1)] \\ \text{[2]} & & +P(Y=1|S_{VH}=0,S_{GH}=1)\times E[P(S_{VH}=0,S_{GH}=1)] \\ \text{[3]} & & +P(Y=1|S_{VH}=S_{GH}=0,S_{GL}=1)\times E[P(S_{VH}=S_{GH}=0,S_{GL}=1)] \\ \text{[4]} & & +P(Y=1|S_{VH}=S_{GH}=S_{GL}=0,S_{VL}=1)\times E[P(S_{VH}=S_{GH}=S_{GL}=0,S_{VL}=1)] \\ \text{[5]} & & +P(Y=1|S_{VH}=S_{GH}=S_{GL}=S_{VL}=0)\times E[P(S_{VH}=S_{GH}=S_{GL}=S_{VL}=0)] \end{matrix}$$

(Eq 14a)

[1]+[2]+[3]+[4] is the fraction of prevalence from genetic contributions

[5] is the fraction of prevalence from other contributions (environmental, unknown)

The ratio between [1]+[2]+[3]+[4] and [5] can be determined by heritability if available. Otherwise, a ratio of 1 is used in this work.

We assume [1]=[2]=0 ($E[P(S_{VH}=1)]=E[P(S_{GH}=1)]=0$) and [3]=[4].

$$E[P(S_{GL}=1)]=\frac{\text{[3]}}{P(Y=1|S_{VH}=S_{GH}=0,S_{GL}=1)}$$

(Eq 14b)

$$E[P(S_{GL}=0,S_{VL}=1)]=\frac{\text{[4]}}{P(Y=1|S_{VH}=S_{GH}=S_{GL}=0,S_{VL}=1)}$$

(Eq 14c)

Assuming S_GL_ and S_VL_ are independent,

$$E[P(S_{GL}=0,S_{VL}=0)]=E[P(S_{GL}=0)]E[P(S_{VL}=0)]=(1-E[P(S_{GL}=1)])(1-\frac{E[P(S_{GL}=0,S_{VL}=1)]}{1-E[P(S_{GL}=1)]})$$

(Eq 14d)

Posterior probabilites of S_VL_ (Eq. 8) and S_GL_ (Eq. 9) are likely to be confounded by ascertainment bias, given the wide range of annotated variants and genes available for different phenotypes (Figure 4). We incorporate two weights $\alpha_{S_{GL}}$ and $\alpha_{S_{VL}}$, computed with numerical optimization, to control this bias.

Derivation:

$$E[P(S_{GL}=1)]=1-\prod_{i} E[(1-P(G_{L_{i}}=1))^{\alpha_{S_{GL}}}]$$

(Eq. 15)

Equate (Eq. 14b) and (Eq. 15)

Solve for $\alpha_{S_{GL}}$.

According to (Eq. 14b) and (Eq. 14c)

$$E[P(S_{VL}=1)]=\frac{E[P(S_{GL}=0,S_{VL}=1)]}{1-E[P(S_{GL}=1)]}$$

(Eq. 16)

$$\begin{matrix} E[P(S_{VL}=1)] & =1-\prod_{i} E[((OR_{i})^{V_{L_{i}}})^{-\alpha_{S_{VL}}}]\text{ } \\ & =1-\prod_{i} \sum_{j\in\{0,1,2\}} (OR_{i})^{-j\alpha_{S_{VL}}}P(V_{L_{i}}=j) \end{matrix}$$

(Eq. 17)

Equate (Eq. 16) and (Eq. 17)

Solve for $\alpha_{S_{VL}}$.

Optimization requires the following constraints for numerical stability:

$$0\leq\alpha_{S_{VL}}\leq1$$

$$0\leq\alpha_{S_{GL}}\leq1$$

(Eq. 18)

To compute $\alpha_{S_{GL}}$ and $\alpha_{S_{VL}}$ in (Eq. 15) and (Eq. 17) requires estimates of expected values for the frequency of functionally impacted low penetrance genes and the odds ratios of GWAS hits associated with the phenotype. We estimated these expected values using databases of variants in general populations, the Exome Variant Server ESP6500 [[2](#_ENREF_2)] and 1000 Genomes Project data [[3](#_ENREF_3)]. Using the ESP6500, we find all rare variants (<1% MAF) in the selected genes and their population frequencies and compute functional impact scores (Eq. 2). Next, for each gene we simulate a population of 10,000 individuals, to match the frequency spectrum of rare variants in ESP6500. We assume that rare variants within a gene are not in linkage disequilibrium. We calculate $P(G_{L_{i}}=1|Data)$ for each simulated individual to estimate (Eq 15). We calculate the allele frequency of each selected GWAS hit in the ESP6500 (for coding variants) and 1000 Genomes (for non-coding variants). We use the allele frequencies and the assumption of Hardy-Weinberg equilibrium, to compute (Eq 17).

For the great majority of variant genotypes, we were unable to find literature or database annotations that estimated penetrance, with respect to the associated phenotypes in our study. However a quantitative measure related to penetrance, the odds ratio, was available for most GWAS hits. We converted odds ratio to penetrance, using estimates of genotype population frequencies and phenotype prevalence, as follows:

The binary random variables *V* and *Y* represent a variant genotype and a phenotype of interest. By definition,

$$OR=\frac{P(V=1|Y=1)/(1-P(V=1|Y=1))}{P(V=1|Y=1)/(1-P(V=1|Y=0))}$$

(Eq. 19)

which we rewrite by setting the numerator to q/(1-q) and the denominator to p/(1-p)

$$OR=\frac{q/(1-q)}{p/(1-p)}=\frac{q-qp}{p-qp}$$

(Eq. 20)

then

$$OR\times p-q=(OR-1)\times qp$$

(Eq. 21)

$$\begin{matrix} P(V=1) & =P(V=1,Y=0)+P(V=1,Y=1) \\ & =P(V=1|Y=0)P(Y=0)+P(V=1|Y=1)P(Y=1) \\ & =p\times P(Y=0)+q\times P(Y=1) \end{matrix}$$

(Eq. 22)

The term *P(V = 1)* represents the population frequency of the variant genotype *V*. We estimate this term by counting how often it occurs in the 1000 Genomes database of human variation. In this work, we used frequencies from the 1000 Genomes European-American population, but estimates could be improved by using a population matched to a particular individual. The term *P(Y = 1)* represents the frequency of the phenotype, or its *prevalence.*  Wherever possible, we estimated phenotype prevalence for each individual, considering her/his age, gender, and self-reported ancestry.

Finally, solving for *q,* the penetrance can be computed with Bayes’ rule:

$$P(Y=1|V=1)=\frac{q\times P(Y=1)}{P(V=1)}$$

(Eq. 23)

The probability that a gene is functionally altered in an individual is:

$$P(G=1|T_{GENE})=\frac{P(T_{GENE}|G=1)P(G=1)}{P(T_{GENE}|G=1)P(G=1)+P(T_{GENE}|G=0)P(G=0)}$$

(Eq. 24)

where $T_{GENE}=-2\times\sum_{i=1}^{N} \ln(p_{i})$ and *p_i_* is the VEST P-value of each variant *i* in the gene.

$P(T_{GENE}|G=1)$ is estimated with simulation, based on empirical data. We assume that a single rare functional variant in a gene is sufficient for the function of that gene’s translated product to be altered. We simulate the distribution of *T_GENE_* in a sample of genes having one rare functional variant and *N-1* benign variants, varying *N* from to 1 to 50. $P(T_{GENE}|G=1)$ is estimated by generating 10,000 functionally altered genes each of which contains one rare functional variant randomly drawn from the HGMD DM class and *N-1* variants randomly drawn from 1000 Genomes (MAF > 0.01). $P(T_{GENE}|G=0)$ is estimated by generating 10,000 genes that are not functionally altered, by randomly drawn *N* variants (MAF > 0.01) from 1000 genomes. We assume a uniform prior.

$$P(G=1)=P(G=0)=0.5$$

(Eq. 25)

**Model assessment.**

We assessed models by their classification performance, as area under the ROC curve (AUC). We computed the statistical significance of AUC with permutation tests as follows. Let ***Y_ij_*** and ***M_ij_*** be two 130x146 matrices, where each row *i* indexes a PGP participant and each column *j* indexes a phenotype. ***Y_ij_*** is a matrix of posterior probabilities, with respect to each PGP participant *i* having phenotype *j.*  ***M_ij_*** is a binary matrix, and each component shows the true status of PGP participant *i* with respect to phenotype *j*  (0 or 1). We calculated the actual AUC for each phenotype *j* by comparing columns ***Y._j_*** and ***M._j_***. Next, we generated matrices ***M_ij_^1^, M_ij_^2^, . . . , M_ij_^K^*** (K=10,000), where each matrix was a random permutation of the rows of ***M_ij_.***  We constructed a null distribution of AUC statistics by calculating the AUC for each phenotype *j* using columns ***Y._j_*** and ***M._j_^1^, M._j_^2^, . . . , M._j_^K^*** . The estimated p-value for phenotype *j* AUC is

(Eq 26)

The null distribution of AUC statistics was also used to compute p-values for each null AUC *k*

(Eq 27)

Let be a list of p-values (Eq 3) for all *L=146* phenotypes, sorted in ascending order. Then for each p-value cutoff (at rank *l*).

(Eq 28)

and

(Eq 29)

This null distribution assumes there is correlation structure among the phenotypes, which should be preserved in permutation testing.

An alternate null distribution can also be generated by randomly permuting the columns of ***Y_ij_***. This null distribution makes the assumption that phenotypes are independent and exchangeable, so that preserving correlation structure is not necessary.

**Rank order matching of PGP participants and phenotypic profiles in CAGI 2012-13.**

For each of the 77 PGP participants in the CAGI challenge, we used their genome sequence as input to models for each of the *J*=243 phenotypes included in the challenge, and the posterior probability of each phenotype was computed. Noting that each phenotype profile consists of *J* components (0 or 1), the match between PGP genome *i* and phenotypic profile *k* can be modeled with a Bernoulli likelihood

$$L_{i,k}=\prod_{j=1}^{J} \left[P(Y_{ij}=1|Data)^{PS_{jk}}P(Y_{ij}=0|Data)^{(1-PS_{jk})}\right]^{w_{j}}$$

(Eq. 30)

where *j* indexes phenotypes, *Y_ij_* is the predicted status of phenotype *j* for PGP genome *i*, *PS_jk_* is the status of phenotype *j* reported in phenotypic profile *k*, and *w_j_* is the weight of our prediction for phenotype *j*. The probability of a match between PGP genome *i* and phenotypic profile *k* is

$$P(i,k)=\frac{L_{i,k}}{\sum_{k} L_{i,k}}$$

(Eq. 31)

The *w_j_*  were estimated using a held-out set of 20 known PGP genome-to-profile pairs that were provided by the CAGI organizers. Briefly, we maximized

(Eq 32)

with a greedy optimization algorithm.

**References**

1. Carter H, Douville C, Stenson PD, Cooper DN, Karchin R (2013) Identifying Mendelian disease genes with the variant effect scoring tool. BMC Genomics 14 Suppl 3: S3.

2. Anonymous NHLBI exome sequencing project (ESP) exome variant server.

3. Genomes Project C, Abecasis GR, Altshuler D, Auton A, Brooks LD, et al. (2010) A map of human genome variation from population-scale sequencing. Nature 467: 1061-1073.

4. Pearl J (1988) Probabilistic reasoning in intelligent systems: networks of plausible inference. San Mateo, Calif: Morgan Kaufmann Publishers, Inc.

5. Cariaso M, Lennon G (2012) SNPedia: a wiki supporting personal genome annotation, interpretation and analysis. Nucleic Acids Res 40: D1308-1312.

6. Bodmer W, Bonilla C (2008) Common and rare variants in multifactorial susceptibility to common diseases. Nat Genet 40: 695-701.
